# Seasonal Variations in Proton Binding Characteristics of Dissolved Organic Matter Isolated from the Southwest Baltic Sea

**DOI:** 10.1021/acs.est.1c04773

**Published:** 2021-11-12

**Authors:** Pablo Lodeiro, Carlos Rey-Castro, Calin David, Jaume Puy, Eric P. Achterberg, Martha Gledhill

**Affiliations:** † GEOMAR Helmholtz Centre for Ocean Research Kiel, Wischhofstraße 1-3, 24148 Kiel, Germany; ‡ Department of Chemistry, University of Lleida—AGROTECNIO-CERCA Center, Rovira Roure 191, 25198 Lleida, Spain

**Keywords:** DOM, nonideal competitive adsorption (NICA), proton binding, acid−base, log *K*_H_, solid-phase extraction

## Abstract

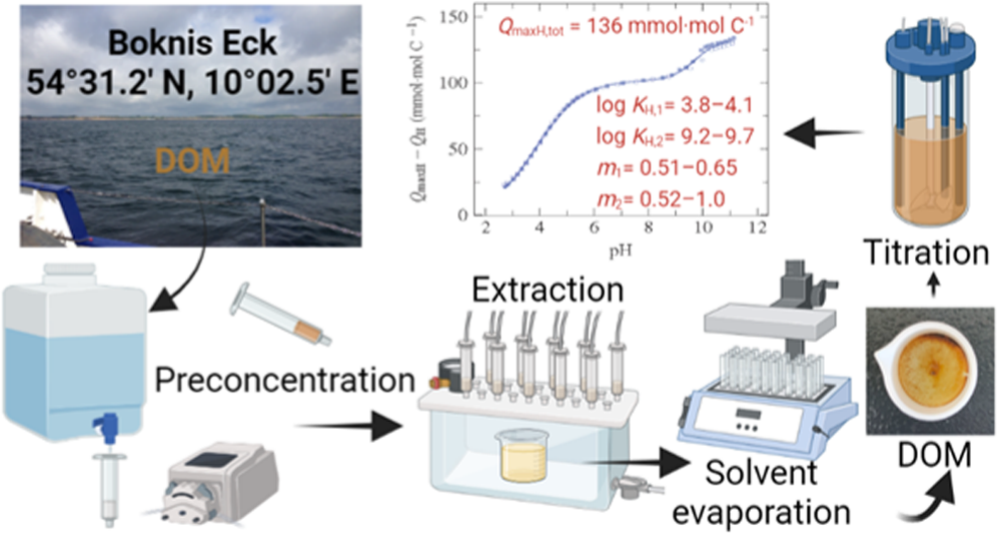

The physicochemical characteristics of dissolved organic matter (DOM) strongly influence its interactions with inorganic species such as protons and trace elements in natural waters. We collected water samples at Boknis Eck, a time series station in the Baltic Sea with a low exposure to freshwater inputs, to investigate how seasonal fluctuations impact the proton binding properties of the isolated DOM. We used potentiometric titrations to assess the binding properties of solid-phase extracted DOM (SPE–DOM) over a seasonal cycle. We report and critically analyze the first NICA parameters estimates of carboxylic-like and phenolic-like sites for brackish water SPE–DOM. The total amount of functional groups (*Q*_maxH,tot_) showed no seasonal fluctuations and an average value of 136 ± 5.2 mmol·mol C^–1^. The average proton affinity (log*K*_H_) and binding site heterogeneity (*m*) showed a relatively minor variability for samples obtained between April and September, when the water remained stratified. These results contribute to a better understanding of the ion binding characteristics of DOM in natural brackish waters.

## Abstract 2

The functional group concentrations, heterogeneity, and thermodynamic constants of proton binding by coastal SPE−DOM can be influenced by environmental seasonal fluctuations.

## 1 Introduction

Dissolved organic matter (DOM) in marine systems acts as a key bioactive carbon reservoir, which holds as much carbon as the atmosphere.^1^ Marine DOM also regulates the chemical speciation of key trace elements that play roles in the ocean as essential nutrients (e.g., Fe) or pollutants (e.g., Pb).^2,3^ The binding of ions depends on water chemistry parameters such as pH, ionic strength, and solution composition, and the properties of DOM, i.e., the availability of the binding sites, their quantity, and accessibility, and on the affinity between chemical species and binding sites.^4^ Protons are always present in solution and compete with other cations (e.g., trace metals) for DOM binding sites. Competitive adsorption effects and the overall DOM binding capacity are therefore related to the acid–base properties (i.e., proton binding) of DOM. For example, the number of binding groups obtained from a potentiometric titration is usually considered a potential maximum of binding sites for species such as metals.^5^ Marine DOM acid–base properties have received little attention to date,^6−8^ despite their key role in nutrient/pollutant binding, DOM–particle interactions, and organic alkalinity.

Current chemical speciation models lack accurate information of the nature of the organic acids contributing to chemical speciation of trace elements and coastal alkalinity, which limits their applicability.^9,10^ Thus, binding models that explicitly link metal complexation to the acid–base properties of organic matter and incorporate heterogeneous distributions of binding sites need to be implemented. Our own work to date has demonstrated that the description of the acid–base properties of marine and brackish water-derived SPE–DOM using the NICA–Donnan model can be used to enhance our understanding of metal biogeochemistry in the contemporary ocean and coastal waters, as well as for predicting changes resulting from various future climate scenarios such as acidification.^6,11^

Although the acid–base properties of DOM have been shown to be useful, there has to date been very few quantifications of binding site concentrations, protonation constants, or binding site heterogeneity for marine SPE–DOM.^6,7,12^ Therefore, we have little knowledge of how variable these binding site properties might be in the marine environment. Littoral zones and coastal seas regulate the yield and storage of carbon at the land–ocean interface. Dissolved organic carbon (DOC) concentrations in coastal systems are influenced by inputs from phytoplankton, rivers, groundwater, and benthic sources as well as anthropogenic sources, with removal by remineralization, microbial activity, and photodegradation.^13^ The carbon cycle in coastal margins is usually dominated by the influx of terrestrial organic matter and nutrients, and the character and behavior of DOM along littoral zones is highly variable. This variability and complex dynamics of DOM have the potential to result in changes in binding properties^10^ and make them ideal environments in which to undertake a first assessment of the potential variability of binding site properties in marinewater DOM.

Semienclosed seas, like the Baltic Sea, are characterized by pronounced fluctuations in pH, oxygen, nutrient concentrations, and salinity.^14^ The physicochemical variations are determined by marine and freshwater inputs, anthropogenic sources, enhanced primary productivity, and soil diversity that influence the DOM residence time, transport, and reactivity.^15^ Baltic waters present typically high concentrations of marine and terrestrial DOM (mg·L^–1^ range) derived from primary production, terrestrial/freshwater inputs, and bio–phototransformation.^16^ Furthermore, the enhanced DOM levels in the Baltic Sea may affect the acid–base water chemistry with consequences for modeling of the carbonate system.^17−19^

Terrestrial DOM has a higher abundance of aromatic compounds, and thus a higher susceptibility to photochemical degradation than marine DOM.^20^ The increase in O/C ratios and aromaticity as DOM ages^21^ is likely to result in changes in the acidity of organic matter and thus the ability of DOM to bind elements and pollutants. Terrestrial and marine DOM may also differ in stoichiometry of C, N, and P, with higher C/N ratios found in soil- and river-derived DOM than in autochthonous DOM produced by phytoplankton.^15^ Moreover, H/C ratios decrease and O/C ratios increase as DOM ages.^22^ The DOM chemical composition is complex, though the general consensus is that carboxylic acids and, to a lower extent, phenolic compounds (ca. 25% of the total) are the two main binding groups in its structure.^23,24^

The physicochemical analysis of DOM is not straightforward and usually requires preconcentration from seawater and isolation techniques.^25,26^ Despite the challenges associated with the analysis of the complex mixtures of compounds, much progress has been made in recent years in characterizing organic matter through absorption spectroscopy and mass spectrometry methods.^27−30^ Though very useful, none of those analyses allow for the examination of competitive interactions between metal ions and protons for binding to functional groups of DOM in natural environments. On the other hand, potentiometric titrations of SPE–DOM provide key physicochemical parameters such as the total amount of active chemical groups, thermodynamic equilibrium constants, and heterogeneity of DOM by studying its acid–base properties. Moreover, potentiometric titrations carried out at different ionic strengths allow us to address the potential impact of pH and salinity changes on the protonation state of the chemical groups present in SPE–DOM, and thus their binding to, e.g., trace elements.

Here, we investigate the physicochemical properties of DOM extracted from surface waters that were collected at Boknis Eck, a time series station located in the Southwestern Baltic Sea. This area is characterized by the periodic inflow of saline North Sea water through the Kattegat and the Great Belt, and low river water inputs. The research covers an annual cycle and hence, potentially resolves seasonal variability. Our investigation also addresses fundamental but unsolved questions concerning the extraction/preconcentration yield, behavior, and dynamics of DOM in estuarine environments.

## 2 Materials and Methods

### 2.1 Seawater Collection

Samples were collected on the 3rd of March (sample 03/03), 28th of April (sample 28/04), 19th of May (sample 19/05), 9th of June (sample 09/06), and 15th of September (sample 15/09) 2020 at the Boknis Eck time series station located in southwest Baltic Sea (54°31.2′ N, 10°02.5′ E). Surface water (ca. 1 m depth) was collected using acid-washed tubing and suction provided by a Teflon bellows pump (Almatec A15). A cellulose acetate membrane filter (Sartobran 300, 0.45/0.2 μm, Sartorius), previously rinsed with >5 L of high-purity water with a resistivity of 18.2 MΩ·cm, was attached to the end of the tubing. Prior to sample collection, the cartridge filter was additionally rinsed with approximately 1 L of seawater. The filter was replaced after filtering 20–30 L of seawater. Water was pumped into acid-cleaned high-density polyethylene (HDPE) and fluorinated HDPE 20 L carboys and acidified with HCl (Romil UHP grade) to a final pH of 2 prior to DOM preconcentration. Sample 03/03 was used to investigate the effect of the extraction volume (10, 20, 30, and 50 L) and flow rate (10, 50, and 200 mL·min^–1^) on DOM proton binding, C/N ratios, and extraction yields.

### 2.2 Dissolved Organic Matter Extraction

Dissolved organic matter was extracted from the collected seawater using solid-phase extraction cartridges (Mega Bond–Elut PPL 5 g, 60 mL, Agilent). The isolated DOM is referred to as SPE–DOM through the text. The PPL cartridges were previously soaked for 12 h with 50 mL of methanol (Fisher Scientific LC–MS grade) and then washed by passing 15 mL of HCl 0.1% v/v through each cartridge before use. Then, 20 L of acidified seawater was pumped through each cartridge. After that, the PPL cartridges were washed with 15 mL of high-purity water and then soaked for 10 min with (2×) 10 mL of acetonitrile to elute the DOM extract. The acetonitrile–DOM solution (20 mL from each cartridge) was collected in a Teflon pot and dried under a stream of ultrapure N_2_ gas. We analyzed the concentration of metals (Al, Fe, Cu, Zn, Ni, Co, and Mn) in the acetonitrile–DOM solution by SEC–ICP-MS (size exclusion chromatography–inductively coupled plasma-mass spectrometry) and observed background values of less than 3 μmol·mol^–1^ C associated with our DOM. The extraction efficiencies were determined as the ratios between the DOC content of the DOM extracts and the DOC in the original water samples.

### 2.3 Seawater Analysis

Data and the analytical methods of analysis for nutrients (phosphate, silicic acid, nitrate, and nitrite), dissolved total oxidized nitrogen (TON), pH, chlorophyll *a*, temperature, and salinity at Boknis Eck are accessible online at http://www.bokniseck.de/. Samples for DOC and dissolved organic nitrogen (DON) were collected from the seawater carboys in precombusted 25 mL borosilicate glass vials. The analysis of DOC and total dissolved nitrogen (TDN) concentrations were done using a Shimadzu TOC-L analyzer equipped with an ASI autosampler. The DON concentrations were calculated from TDN by subtraction of nitrate and nitrite concentrations.

### 2.4 Dissolved Organic Matter Stock Solutions

The solid extracts were dissolved in NaOH (0.02 M, extrapure, 98%, Acros Organics) to a final SPE–DOM concentration between 1.2 and 5.2 g·L^–1^ and preserved in the dark at 4 °C. These SPE–DOM stock solutions were used, usually within the first week of preparation, to obtain the samples for titration as described below.

### 2.5 Potentiometric Titrations

Two automatic titration systems operated using Matlab scripts were used for the titration experiments. Each system consisted of a burette (Metrohm, Dosimat 765) with a capacity of 10 mL and a benchtop pH meter (Thermo Orion, 720Aplus), both connected to a computer. The electromotive force was monitored with a combined glass electrode (Orion ROSS Ultra 8102BNUWP) and recorded using a drift criterion of <0.05 mV·min^–1^, with a maximum stabilization time of 120 min. The electrodes were filled with a 0.7 M NaCl solution to match the ionic strength of the titrated samples. The electrodes were calibrated before and after each SPE–DOM titration, on the hydrogen-ion concentration scale^31^ for NaCl 0.7 M by titrating HCl with NaOH, using the same potential drift criterion and temperature (25 °C) as in the titration experiments. Absorption of CO_2_ from ambient air was minimized by preparing the titrant solutions with high-purity water previously boiled under constant N_2_ purge and storing them in sealed bottles fitted to soda lime traps. The residual concentration of dissolved carbonates, checked periodically through Gran titrations,^32^ was lower than 1%.

Duplicate titrations were performed in deaerated samples degassed by bubbling with water-saturated nitrogen in a sealed thermostated vessel containing 20 mL of a diluted SPE–DOM solution. The titrated solutions were prepared by diluting an SPE–DOM stock with 4 M NaCl (puriss. p.a., ≥99.5%, Merck), ∼0.1 M HCl (Optima Grade, Fisher Scientific), and high-purity water to final concentrations of 0.86–1.47 g DOM·L^–1^ (456–715 mg C·L^–1^). The HCl solution was previously standardized by titration with sodium tetraborate decahydrate (borax, ReagentPlus, ≥99.5%, Sigma-Aldrich).

The ionic strength (I) of the titrated solutions was fixed to 0.7 M using NaCl as inert supporting electrolyte. The initial solution pH was fixed to a value of ca. 3.0. The temperature was kept at 25 ± 0.1 °C using a controlled temperature bath circulator (Thermo Scientific, A10). Sodium hydroxide (extrapure, 98%, Acros Organics) (0.1 M), previously standardized with potassium hydrogen phthalate (puriss. p.a., ≥99.5%, Sigma-Aldrich), was added using the burette at variable volume intervals to perform the calibration and titration experiments. A typical SPE–DOM titration experiment took about 6–8 h, including an initial solution equilibration step under N_2_ bubbling for ca. 1 h.

### 2.6 Theory and Calculations

#### 2.6.1 NICA Model

The proton titration data were described by the bimodal NICA isotherm.^5,33^ In the absence of metal cations able to compete with protons for the specific binding to the functional groups of DOM (monocomponent system), the bimodal NICA isotherm is formally identical to the weighted sum of two Langmuir–Freundlich isotherms^34^
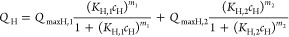
1where *Q*_H_ stands for the amount of bound protons per mol of DOC (mmol·mol C^–1^), *Q*_maxH,*j*_ is the total amount of titratable proton sites within each distribution, *K*_H,__*j*_ is the median value of the *j*th affinity distribution for protons, *c*_H_ is the proton concentration, and *m*_*j*_ (0 < *m*_*j*_ ≤ 1) is a parameter related to the width of the affinity distribution function (a measure of the apparent binding heterogeneity). The limiting value of *m*_*j*_ = 1 corresponds to a perfectly homogeneous set of sites. The subindexes *j* = 1 and 2 represent the most and less acidic modes in the affinity distribution of sites, usually associated with carboxylic and phenolic functional groups, respectively.

Note that the NICA isotherm was used without any electrostatic model to account for the polyelectrolytic effect and, therefore, the fitted binding parameters must be regarded as conditional values for the ionic strength of the experiments (0.7 M). A small electrostatic contribution to the binding is expected at the enhanced ionic strength of the experiments. Moreover, the uncertainty in the extrapolation of the results of this work to actual seawater conditions should be highlighted since the competition effect of major cations has not been assessed.

#### 2.6.2 Strategy for the Derivation of NICA Model Parameters

The experimental datasets of titrant volume and pH were converted into pH and charge curves using mass and charge balance relationships,^6^ as detailed in the Supporting Information. Note that pH was measured on the hydrogen-ion concentration scale, as described in Section 2.5. The optimization of the NICA parameters was carried out by nonlinear regression using MATLAB to minimize the root-mean-square error (RMSE, in mmol·mol C^–1^) in the DOM charge
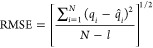
2where *q_i_* and *q̂*_*i*_ are the experimental and fitted values of DOM charge, *N* is the number of data points, and *l* is the number of model parameters. We used the MATLAB function “lsqcurvefit”, which is based on the Levenberg–Marquardt algorithm, the most widely used optimization algorithm.

The conditional values estimated at *I* = 0.7 M from the generic (intrinsic) fulvic acid (FA) parameters of Milne et al.^35^ were calculated taking into account the ratio between the local concentration of protons in the fulvic macromolecular domain and their concentration in bulk solution, using a suitable value of the Donnan factor (see further details in the Supporting Information). Note that we used NaCl as background electrolyte while FA parameters of Milne et al. were obtained in several 1:1 electrolyte solutions.^35^

## 3 Results and Discussion

### 3.1 Bulk Brackish Water DOM

Water sample 03/03, collected at the beginning of March, presented higher salinity (21.679) and dissolved oxygen (11.971 mg·L^–1^) values than all of the other samples collected in the same year until mid-September (Table S1). This indicates an influence of this water sample from the North Sea, which enters the Eckernförde Bay as a dense bottom current and mixes with the deep waters at Boknis Eck while replenishing depleted oxygen levels.^36,37^ In February 2020, dominant southwesterly winds caused the surfacing of the North Sea mixed water at Boknis Eck with increased nutrient concentrations and the subsequent chlorophyll maximum (Table S1). From mid-March until mid-September, the water column at Boknis Eck was strongly stratified, which together with the enclosed nature of the Bay and the limited freshwater inputs^36^ resulted in a nearly constant surface salinity of 14.7 ± 0.2.

Surface DOC measured between March and mid-September showed an increasing trend from 217 to 381 μmol C·L^–1^ (Table S1). Riverine and terrestrial runoff inputs into waters near Boknis Eck are reported to be not significant.^36^ We therefore hypothesize that DOC accumulated over the course of the season was due to extracellular release, cell lysis, and excretion by grazers, phytoplankton, virus, or prokaryotes, and the remineralization of particles.^38^ In contrast, DON values remained approximately constant during spring and summer (12.5–15.7 μmol·L^–1^), with a lower value (8.5 μmol·L^–1^) for sample 03/03. As a result, we measured enhanced values of C/N molar ratios in March and September, and constant values between April and June. C/N molar ratios in the water samples thus showed a positive correlation (*r*^2^ = 0.8476, *n* = 4) with chlorophyll *a* data, which is suggestive of a link between DOM stoichiometry and phytoplankton abundance.

**Figure 1:**
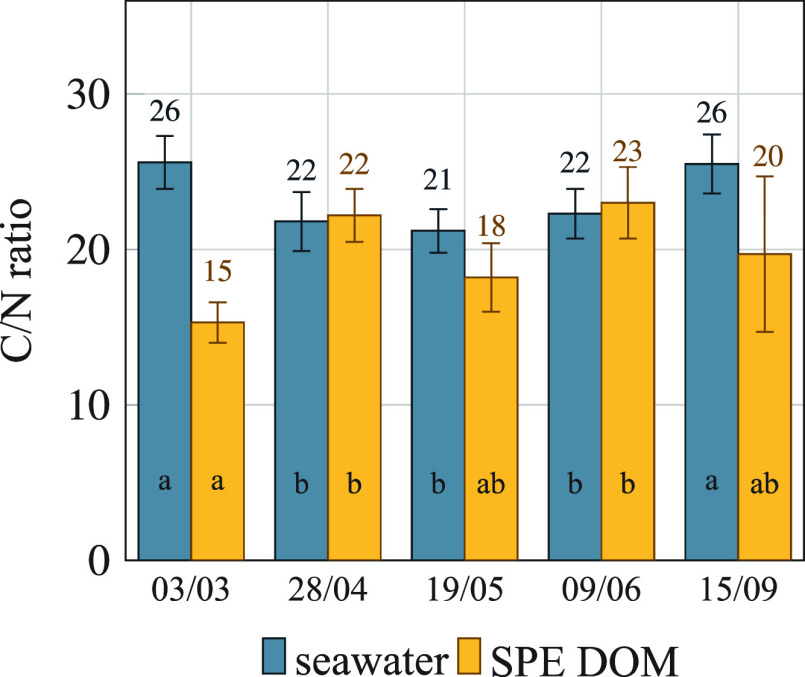
C/N molar ratios for seawater (blue bars) and SPE–DOM (yellow bars) measured along the year 2020 (the *x*-axis tick labels indicate the sampling date). Bar heights (and the numbers above them) correspond to the mean values, and error bars indicate the standard deviation. Statistically indistinguishable means are labeled with the same letter (analysis of variance, ANOVA, and least significant difference test (LSD) *P* ≤ 0.05, *n* = 4).

In our samples, the C/N molar ratio (Figure 1) was well above the Redfield ratio of 6.625, in agreement with values previously reported.^39−41^ The first sample collected in March presents enhanced C/N values. This feature likely reflected mixing of DOM associated with inflowing waters from the North Sea (36.8–120.1 μmol C·L^–1^ and 3.5–11.7 μmol N·L^–1^),^42^ and DOM from the Southwest Baltic Sea (198–683 μmol C·L^–1^ and 12–36 μmol N·L^–1^)^43,44^ (Table S1 and Figure 1).

The C/N molar ratio then decreased by the following month (April) to 22. This decrease occurred after the spring phytoplankton bloom as autochthonous DOM is remineralized. The resulting DOM presents constant C/N values between April and June, due to proportional changes in DOC and DON values. Finally, the C/N molar ratio increased from June, gradually approaching that of the DOM in March (26, sample 15/09) as shown in Figure 1.

The relative proportions of labile and inert (recalcitrant) fractions that present different C/N molar ratios are known to vary seasonally, and also with depth.^41,45,46^ Dissolved organic matter should therefore be considered as a continuum of pools with different lability. Seawater bulk properties therefore suggest that the DOM pool changed over our sampled period as a result of changes in water mass properties, phytoplankton productivity, bacterial mineralization, and photochemical degradation. Here, we assess whether these processes have an impact on the SPE–DOM proton binding properties.

### 3.2 Solid-Phase Extracted DOM

#### 3.2.1 Effects of the SPE–DOM Preconcentration Volume and Flow Rate

We used sample 03/03 to investigate the effect of the preconcentration volume and flow rate during the DOM isolation on: (i) DOC extraction efficiency; (ii) percentage of carbon in the extracted DOM; (iii) C_SPE_/N_SPE_ ratios; and (iv) SPE–DOM NICA binding parameters. We then used the selected optimal volume (20 L) and flow rate (200 mL·min^–1^) to extract the DOM from water samples 28/04 to 15/09 and assess seasonal changes in their acid–base properties.

**Figure 2:**
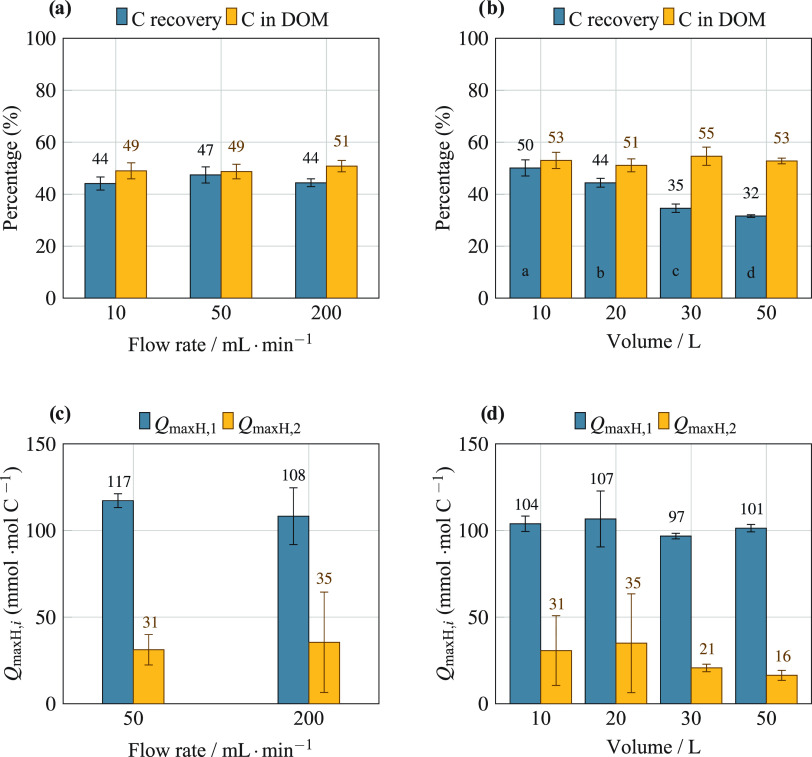
Percentage of carbon recovery (blue bars) and carbon content in SPE–DOM (yellow bars) at different preconcentration flow rates (a) and volumes (b). Bottom panels show the total amount of titratable proton sites within the carboxylic (*Q*_maxH,1_) and phenolic (*Q*_maxH,2_) distributions at different preconcentration flow rates (c) and volumes (d). Bar heights (and numerical values above them) indicate the mean, and error bars indicate the standard deviation. Statistically indistinguishable means are labeled with the same letter (analysis of variance, ANOVA, and least significant difference test (LSD) *P* ≤ 0.05, *n* = 4).

A one-way ANOVA comparison test showed that the mean percentages of DOC recovery (Figure 2a) were not significantly different over the range of flow rates tested (10, 50, and 200 mL·min^–1^). However, the differences in percentage DOC recovery between the volumes investigated (10, 20, 30, and 50 L) were indeed significant (Figure 2b).

These differences arose from an overloading of the columns and DOM breakthrough as the DOM/PPL mass ratio increased.^26^ Accordingly, we observed a negative linear correlation between DOC recoveries and extraction volumes (*r*^2^ = 0.8792, *n* = 4). Maximum DOC extraction efficiency was achieved for a seawater preconcentration volume of 10 L (Figure 2b). Nevertheless, we selected a volume of 20 L as a trade-off between the total amount of DOM extracted and an excessive use of PPL cartridges. Despite the decrease in DOC recovery observed with the sample volume, the mean values of carbon percentages in the SPE–DOM were not significantly affected by either flow rate or volume values studied (Figure 2a,b). We calculated a mean value of 50 ± 2.5% of C content for the SPE–DOM (sample 03/03).

The C_SPE_/N_SPE_ ratios changed significantly with the volume but not with extraction flow rate (Figure S1). We applied a least significant difference test (*P* = 0.05) to the means of C_SPE_/N_SPE_. The results indicate that volumes of 10, 20, and 50 L yielded statistically equivalent results, while only 30 L volume showed significantly larger C_SPE_/N_SPE_ ratios compared to all of the other tested conditions, though no clear explanation can be provided for the observed difference.

**Figure 3:**
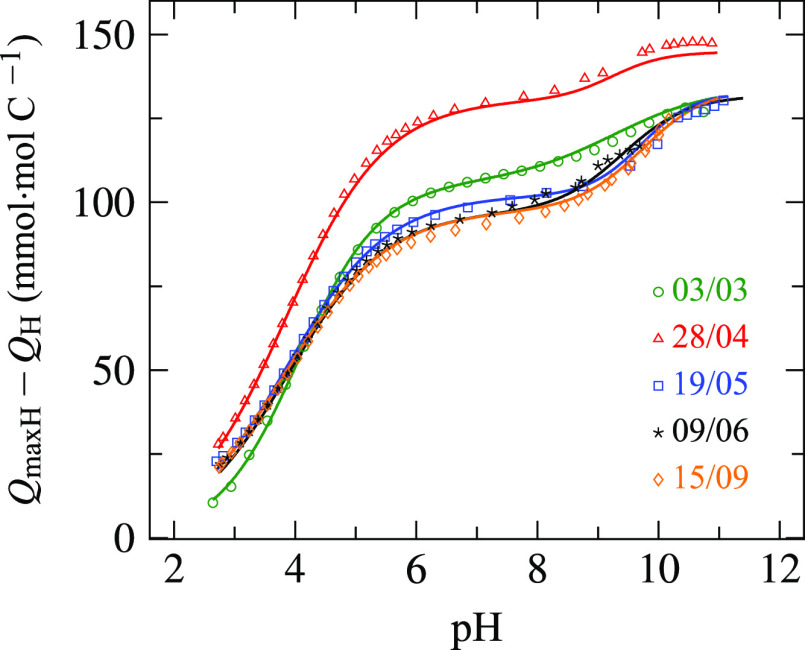
NICA fits to proton titration data of Boknis Eck SPE–DOM at *I* = 0.7 M (NaCl) and 25 °C, for the samples collected in March (green), April (red), May (blue), June (black), and September (orange). Symbols: experimental values from only one of the replicate titrations (for clarity reasons, the complete dataset is only listed in the Supporting Information); lines: model fits.

We fitted the experimental acid–base titration curves of the SPE–DOM at 25 °C with the NICA model for proton ions within the range 2.7–3.0 ≤ pH ≤ 10.5–11.0 (Figure 3). We could not obtain titration data at pH > 10 for the sample extracted at a flow rate of 10 mL·min^–1^, so the corresponding results were not used in the analysis. This is partly due to an insufficient number of phenolic groups in the titration solution, which produced large uncertainties in the charge values at high pH, and also influenced the obtained parameters for the first binding mode. The obtained NICA parameters for sample 03/03 did not vary with extraction flow rates (50 and 200 mL·min^–1^) or volumes (20, 30, 40, and 50 L) studied (Figures 2c,d and S2).

#### 3.2.2 Seasonal Effect on C_SPE_/N_SPE_ Molar Ratios, DOC Recovery, and Carbon Content

The extracted samples showed similar C_SPE_/N_SPE_ values from March as observed for the seawater samples (Figure 1). In addition, the C_SPE_/N_SPE_ molar ratios of samples 03/03, 19/05, and 15/09 are not significantly different. Our values showed marked similarity to those obtained in other studies using PPL cartridges in different marine waters.^26,47^ We also observed that our extracts are not significantly different from the sampled seawater in terms of C/N ratios, except for the sample collected in March (Figure 1). Other authors have associated these differences with an inefficient extraction of more polar organic compounds (e.g., DON) compared with DOC for SPE–DOM.^22,47,48^ The seawater sample 03/03 presents DON values ca. 40% lower than those obtained for the rest of the series (8.5 compared to an average of 14.7 ± 1.6 μmol·L^–1^, for samples from April to September). Since the 03/03 sample had higher salinities and was taken after winter mixing and before water column stratification had set in, we suggest the relatively low DON concentration was likely a signature of the DOM originating from both the North Sea and the Southwest Baltic Sea.

The DOC recovery from the extracted seawater samples decreased only slightly over the season from a maximum value of 45 ± 2.3% in March to a minimum of 37 ± 2.7% in September (Figure S3a). The C content of the SPE–DOM samples also showed small differences, with enhanced values in March, June, and September (51% in average), while for the DOM extracted in April and May, the C content was 43–45% of the extracted DOM mass (Figure S3b).

#### 3.2.3 Acid–Base Properties of Seasonal SPE–DOM

The titrations of seasonally extracted DOM were performed at least in duplicate using two different potentiometric systems. The combined dataset gathered from the replicate titrations of each sample were fitted using the NICA model (Figure 3) to obtain the reported mean parameter values, except for sample 03/03 where the dataset gathered from titrations done with SPE–DOM preconcentrated at different flow rates (50 and 200 mL·min^–1^) and volumes (10, 20, 30, and 50 L) was used.

The confidence intervals for the NICA parameters (samples 28/04 to 15/09, Table S2) were calculated as the difference between independent fits to each individual replicate dataset. For the NICA fitting of sample 03/03, we pooled together the titration datasets obtained at different extraction flow rates and volumes (see Section 3.2.1) and then calculated the standard deviation for each NICA parameter (Table S2) of all of the individual data fits.

The NICA model described accurately the experimental proton binding curves of each of the SPE–DOM samples with RMSE values <2.2 mmol·mol C^–1^, except for sample 03/03 that included titrations of seven different SPE–DOM extracts. We characterized the first and second groups of binding sites that correspond to the low- and high-affinity distributions, respectively. The data fit was always better for the carboxylic groups of the first mode than for the phenolic functional groups. The higher uncertainty associated with the NICA parameters of the second mode is due to the low concentration of the phenolic groups, and the difficulty of obtaining precise proton concentrations measurements above pH 10 when using glass electrodes due to the extremely low concentration of protons.^35^

**Figure 4:**
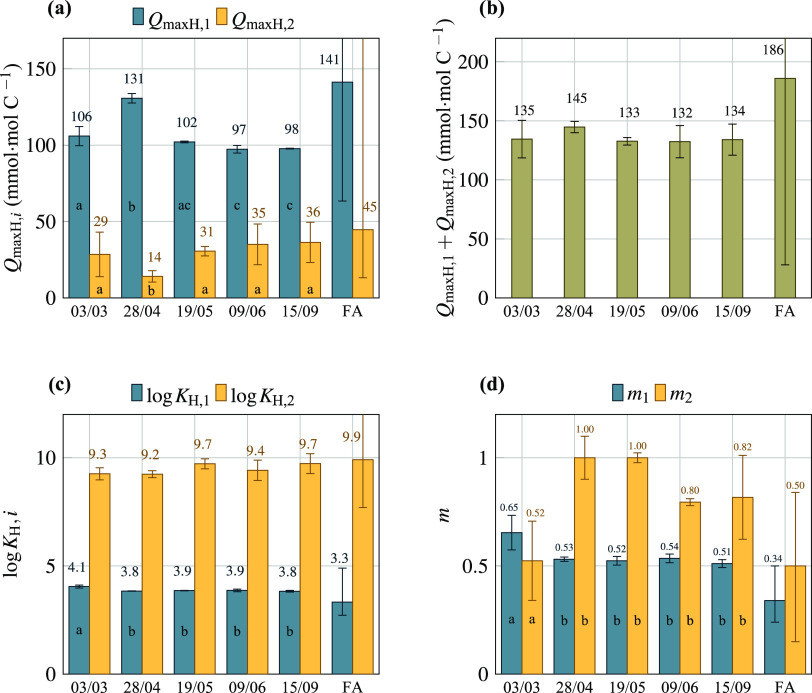
NICA parameters obtained from the fits to proton titration data shown in Figure 3 for the carboxylic (blue bars) and phenolic (yellow bars) distributions: *Q*_maxH,i_ (a), *Q*_maxH,tot_ (b), log *K*_H_ (c), and *m* (d); error bars indicate the standard deviation. The values FA indicate the generic fulvic acid NICA parameters from Milne et al. calculated at *I* = 0.7 M (see Section 2 for details); error bars indicate the range of values for the datasets analyzed: in (a), the values out of the scale are +69 (*Q*_maxH,1_) and +142 (*Q*_maxH,2_); in (b), +84 (*Q*_maxH,tot_); and in (c), +2.6 (log *K*_H,2_). Statistically indistinguishable means are labeled with the same letter (analysis of variance, ANOVA, and least significant difference test (LSD) *P* ≤ 0.05, *n* = 2).

The number of titratable groups (*Q*_maxH,1_ and *Q*_maxH,2_) showed significant differences between all of the samples taken over the season (Figure 4). Nevertheless, the total number of binding sites (*Q*_maxH,1_ + *Q*_maxH,2_) showed no seasonal fluctuations and an average value of 136 ± 5.2 mmol·mol C^–1^. This value suggests that 100 moles of carbon in brackish waters DOM contain 13.6 moles of proton binding sites (13.6% of carbon atoms provides a mol of binding sites), which is in agreement with the range of values (10.8–16.6%) calculated from the data of Huzienga and Kester^12^ and with the fraction of DOC considered to contribute to organic alkalinity (0.12–0.14) in the Baltic Sea, obtained by Ulfsbo et al.^17^ and Kuliński et al.^8^

As expected, the number of carboxylic groups is higher than the phenolic functionalities. The *Q*_maxH,1_ value for sample 03/03 is 106 ± 6.3 mmol·mol C^–1^, while the observed values for the stratified DOM, which correspond to samples from 28/04 to 15/09, ranged from a maximum of 131 ± 3.1 mmol·mol C^–1^ in April (post–bloom) to an average of 99 ± 2.7 mmol·mol C^–1^ between May and September. These values are lower than that described for a generic fulvic acid (ca. 141 mmol·mol C^–1^, considering FA is 50% C by weight) but similar to those previously obtained for DOM extracted in the Kiel Baltic Fjord (103 mmol·mol C^–1^).^6,35^ Despite the unexpectedly low *Q*_maxH,2_/*Q*_maxH,1_ ratio (0.11) obtained for sample 28/04, the average ratio for all of the other samples (0.33 ± 0.05) is in agreement with values previously reported.^35^

We observed clear differences between sample 03/03 and all of the others regarding the values of the binding constants (log *K*_H,1_) and heterogeneity (*m*_1_) of the carboxylic groups, whereas the variations of these parameters among samples 28/04 to 15/09 (Figure 4) were not significant. The observed trend in the carboxylic parameters is in agreement with our hypothesis of the presence of older DOM characteristic of the more saline water mass in March that changes over the year in response to biotic and abiotic processes enhanced by strong stratification of the water column and limited freshwater inputs.

The stratified-DOM (samples 28/04 to 15/09) median values of the first affinity distribution for protons and heterogeneity are 3.86 ± 0.02 and 0.528 ± 0.01, respectively. The obtained values indicate that carboxylic functionalities in stratified biologically active surface waters with higher DOC concentrations are more heterogeneous and slightly more acidic than those observed for the mixed water mass (sample 03/03). When the average April–September values obtained for Boknis Eck are compared with those obtained for DOM extracted from the Kiel Fjord (log *K*_H,1_ = 3.86 and *m*_1_ = 0.56, at 25 °C and *I* = 0.7 M NaCl),^6^ we observed a very similar proton binding constant and a slightly lower heterogeneity of the latter, which is in agreement with the similar salinity (16), season (June–July), and characteristics of the enclosed bay where the Kiel Fjord sample was taken.^6^ In contrast, the carboxylic mode of a generic fulvic acid from freshwater and terrestrial origin presents average values of log *K*_H,1_ = 3.3 and *m*_1_ = 0.34 (calculated from intrinsic NICA parameters at *I* = 0.7 M, as described in Section 2.6). This work confirms therefore our previous finding that carboxylic-like groups in brackish waters SPE–DOM are less acidic and less heterogeneous than in generic terrestrial DOM.

Regarding the phenolic mode, the comparison of the proton binding parameters among the samples is hindered by the relatively large uncertainty of the model fits at a high pH and, therefore, no robust conclusions can be derived. Nevertheless, we observed clear differences in the phenolic heterogeneity parameter of sample 03/03 compared to the remaining samples (Figure 4d), with an *m*_2_ value (0.52 ± 0.2) showing a clearly more heterogeneous second mode, compared to the stratified-DOM values. Furthermore, the *m* value for the second mode of the mixed water (sample 03/03) is similar to the theoretical values calculated for a generic fulvic acid,^35^ in contrast to those observed for the stratified-DOM samples, and carboxylic groups that present clearly more homogeneous *m* values (Figure 4d). Interestingly, the log *K*_H,2_ values are very similar for the mixed, stratified, and generic FA samples (Figure 4c).

Since our previously reported SPE–DOM NICA constants for phenolic groups were based on those estimated at *I* = 0.7 M using the generic intrinsic NICA parameters for fulvic acid (log *K*_H,2_ = 9.41, *m*_2_ = 0.49),^6^ the values presented here represent the first estimates of phenolic group NICA parameters for brackish water SPE–DOM. We therefore report here, for the first time, a full set of NICA constants of SPE–DOM for both phenolic- and carboxylic-type groups (Table S2 with *Q*_maxH,i_ expressed in mmol·mol C^–1^, and Table S3, in mmol·g DOM^–1^).

Although differences in the proton binding parameters along the time series are indeed subtle, we may draw the following tentative conclusion about the general trend observed. It seems that, at the onset of spring, the values of carboxylic acid site density increase slightly by ca. 25% while the phenolic-type sites drop by 52%, and then both recover to the values shown in March. As a result, the total amount of binding sites showed no seasonal fluctuations. In parallel, the carboxylic groups of SPE–DOM become, on average, slightly more acidic and heterogeneous. This behavior might be related to the release of fresh, labile DOM during the phytoplankton bloom which initially adds to the aged mixed water at Boknis Eck, and then become progressively transformed throughout the year. This fresh fraction would be poorer in titratable groups, relatively more heterogeneous in its carboxylic composition but less complex in its phenolic content, and on average, more acidic, compared with the resilient DOM present in the mixed water mass in March. In any case, the combined pool of DOM extracted from brackish waters still seems to show a somewhat lower heterogeneity (in its carboxylic acidity) than an average fulvic acid of terrestrial origin.

## Supporting Information Available

Boknis Eck seawater characteristics (Table S1); SPE–DOM NICA parameters (Tables S2 and S3); C_SPE_/N_SPE_ molar ratios (Figure S1), NICA parameters, log *K*_H_, and *m* (Figure S2), at different DOM preconcentration flow rates and volumes; seasonal changes in carbon recovery and carbon content in SPE–DOM (Figure S3); representative example of the instrumental reproducibility of SPE–DOM titration experiments (Figure S4); calculation of charge–pH curves and conditional parameters of the NICA model (pages S9–S12); and comparison with a generic fulvic acid (page S13) (PDF)
Complete experimental dataset (XLSX)

## Notes

The datasets generated and/or analyzed during the current study are available in the attached spreadsheet.
