# Supplementary material for: Seasonal Variations in Proton Binding Characteristics of Dissolved Organic Matter Isolated from the Southwest Baltic Sea
Source: Environ Sci Technol. 2021 Nov 12;55(23):16215–23. doi: 10.1021/acs.est.1c04773 (PMC8719755; doi:10.1021/acs.est.1c04773)
Supplement: Supplementary file 1 — es1c04773_si_001.pdf [file es1c04773_si_001.pdf]

Seasonal variations in proton binding characteristics of dissolved organic matter isolated from the Southwest Baltic Sea

Pablo Lodeiro[a,b*], Carlos Rey-Castro[b], Calin David[b], Jaume Puy[b], Eric P. Achterberg[a] and Martha Gledhill[a]

[a]GEOMAR Helmholtz Centre for Ocean Research Kiel, Wischhofstraße 1-3, 24148 Kiel, Germany.

[b]Department of Chemistry, University of Lleida – AGROTECNIO-CERCA Center, Rovira Roure 191, 25198, Lleida, Spain.

*Corresponding Author: pablo.lodeiro@udl.cat

Contains 15 Pages, 3 Tables and 4 Figures.

Table S1. Boknis Eck seawater main characteristics (temperature, salinity, pH and oxygen), dissolved organic carbon (DOC), dissolved organic nitrogen (DON) content and chlorophyll–a (Chl–a).

Sample	DOC $\mu mol \cdot L^{-1}$	DON $\mu mol \cdot L^{-1}$	Chl–a $\mu g \cdot L^{-1}$	T ºC	Salinity	pH	O_2 $mg \cdot L^{-1}$
03/03	217 ± 4.4	8.5 ± 0.6	4.46	5.25	21.679	8.011	11.971
28/04	271 ± 13	12.5 ± 1.1	1.55	10.02	14.760	7.996	11.031
19/05	331 ± 16	15.7 ± 1.1	1.51	11.32	14.434	8.079	10.417
09/06	334 ± 22	15.1 ± 1.1	1.27	14.02	14.841	8.128	9.981
15/09	381 ± 18	15.0 ± 1.2	2.96	17.33	14.652	8.189	9.291

Table S2. NICA parameters obtained from potentiometric titrations at I= 0.7 M in NaCl and 25 ºC, from SPE–DOM samples collected at Boknis Eck. The uncertainty of the parameters represents the standard deviation of titrations of DOM extracted at different flow rates (50 and 200 mL·min^{-1}) and volumes (10, 20, 30 and 50 L) for sample 03/03, or the confidence intervals determined from individual fits to the NICA model of titration data for two replicates.

	03/03	28/04	19/05	09/06	15/09
$Q_{maxH,1}$ mmol·mol C^{-1}	106 ± 6.3	131 ± 3.1	102 ± 0.6	97 ± 2.5	98 ± 0.2
log $K_{H,1}$	4.05 ± 0.07	3.84 ± 0.01	3.86 ± 0.01	3.88 ± 0.05	3.83 ± 0.02
m_1	0.65 ± 0.08	0.53 ± 0.01	0.52 ± 0.02	0.53 ± 0.02	0.51 ± 0.01
$Q_{maxH,2}$ mmol·mol C^{-1}	29 ± 14.6	14 ± 3.7	31 ± 3.1	35 ± 13.3	36 ± 13.2
log $K_{H,2}$	9.3 ± 0.3	9.2 ± 0.2	9.7 ± 0.2	9.4 ± 0.2	9.7 ± 0.2
m_2	0.52 ± 0.2	1.0 ± 0.1	1.00 ± 0.09	0.88 ± 0.22	0.87 ± 0.10
RMSE mmol·mol C^{-1}	8.025	2.170	1.348	1.314	2.139

Table S3. Total amount of functional groups of each class ($Q_{\mathrm{maxH,i}}$), expressed per mass of dissolved organic matter (DOM), obtained from potentiometric titrations at I= 0.7 M in NaCl and 25 ºC, from SPE–DOM samples collected seasonally at Boknis Eck. The uncertainty of the parameters represents the standard deviation of titrations calculated using DOM extracted at different flow rates (50 and 200 mL·min^{-1}) and volumes (10, 20, 30 and 50 L) for sample 03/03, or the confidence intervals determined from individual fits to the NICA model of titration data for two replicates.

	03/03	28/04	19/05	09/06	15/09
$Q_{\mathrm{maxH,1}}$ mmol·g DOM^{-1}	4.4 ± 0.3	4.7 ± 0.1	3.83 ± 0.02	4.2 ± 0.1	4.28 ± 0.01
$Q_{\mathrm{maxH,2}}$ mmol·g DOM^{-1}	1.2 ± 0.6	0.50 ± 0.13	1.2 ± 0.1	1.5 ± 0.6	1.6 ± 0.6

Figure S1. C/N molar ratios for solid–phase extracted dissolved organic matter (blue bars) from seawater preconcentrated at different flow rates and volumes (sample 03/03). Bar heights (and the numbers above them) correspond to the mean values, and error bars indicate the standard deviation. Statistically indistinguishable means are labelled with the same letter (analysis of variance, ANOVA, and least significant difference test (LSD) $P \leq 0.05$, $n = 3$).

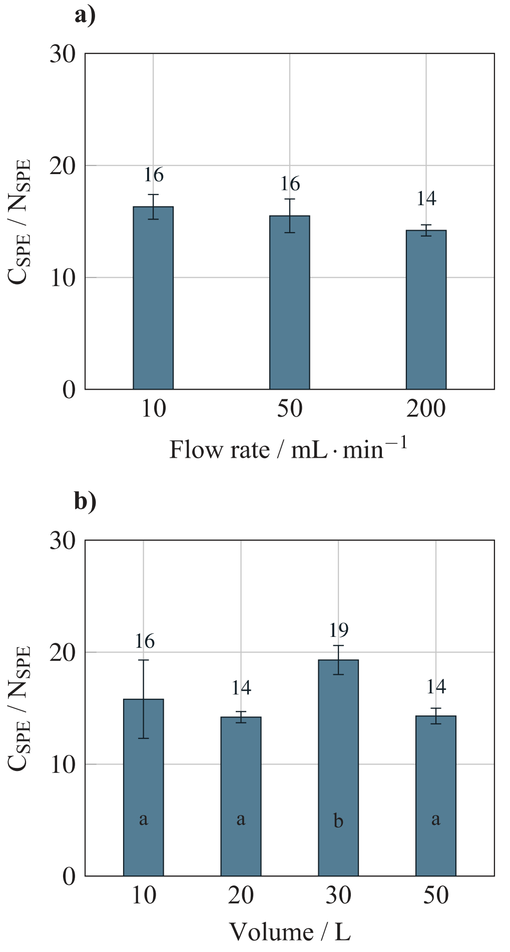

Figure S2. NICA parameters for the carboxylic (blue bars) and phenolic (yellow bars) distributions of the median value affinity distribution for protons, log K_H, (upper panels), and the apparent binding heterogeneity, *m*, (lower panels), at different preconcentration flow rates (a–c) and volumes (b–d). Bar heights (and numerical values above them) indicate the mean, and error bars indicate the confidence intervals calculated as the difference between independent fits to each individual replicate dataset (n = 2).

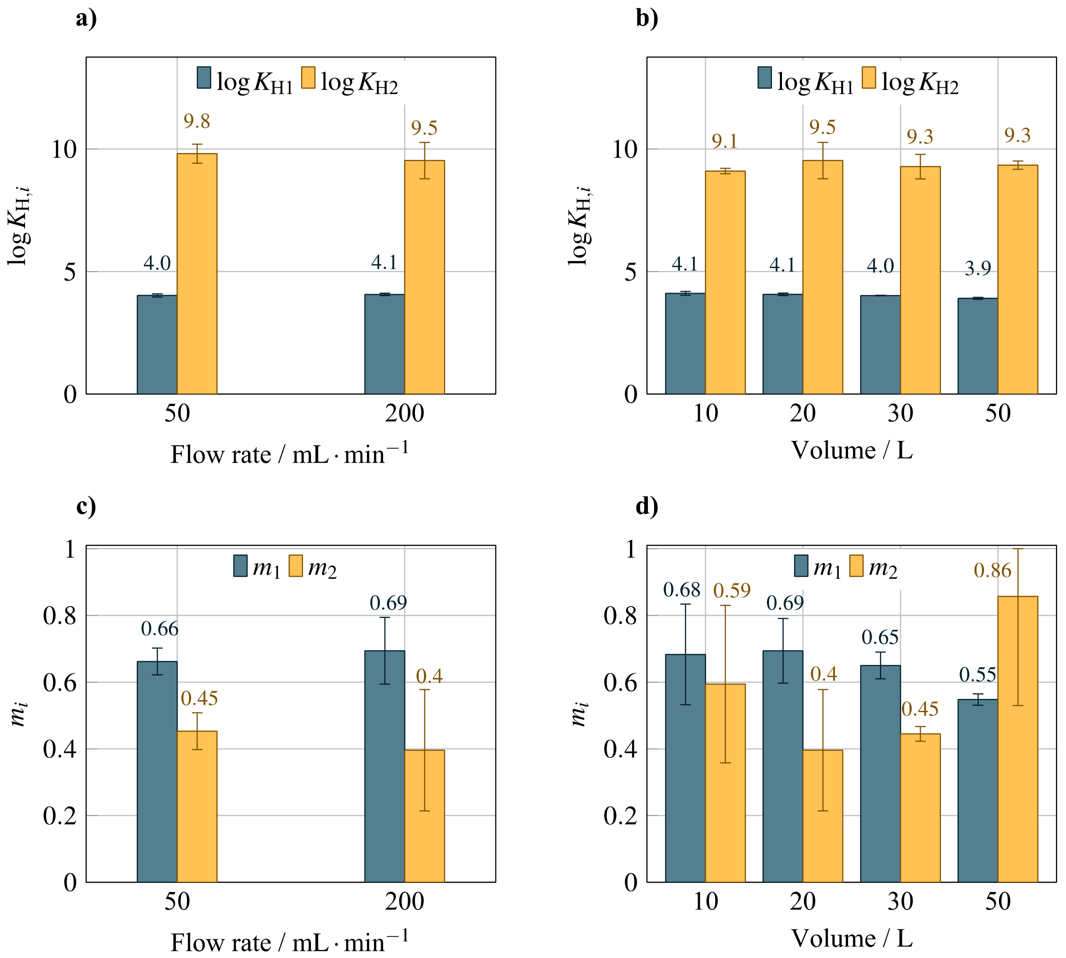

Figure S3. Percentage of carbon recovery (a) and carbon content in extracted DOM (b) measured along the year 2020 (the x-axis tick labels indicate the sampling date). Bar heights (and the numbers above them) correspond to the mean values, and error bars indicate the standard deviation. Statistically indistinguishable means are labelled with the same letter (analysis of variance, ANOVA, and least significant difference test (LSD) P ≤ 0.05, n = 4).

a)

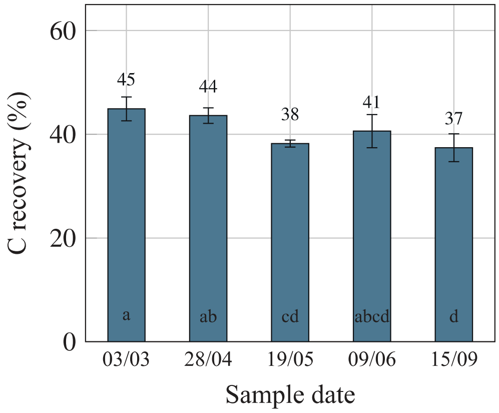

b)

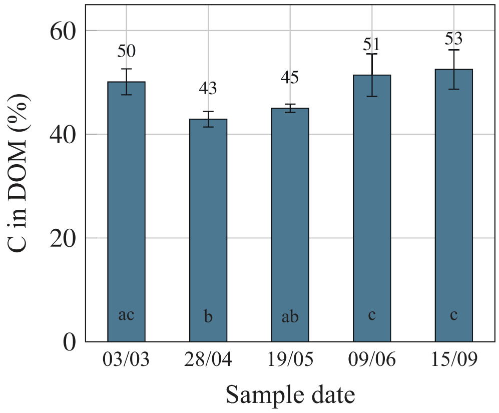

Figure S4. A representative example of the instrumental reproducibility of SPE–DOM titration experiments: the open and filled symbols correspond to two replicate titrations of Boknis Eck SPE–DOM at I= 0.7 M (NaCl) and 25 °C, for the samples collected the 19th May (sample 19/05). Each experiment was performed in a separate potentiometric setup. Only one of these charge curves is shown in Figure 3 for clarity reasons. The solid line corresponds to the NICA isotherm fitted to the combined data set.

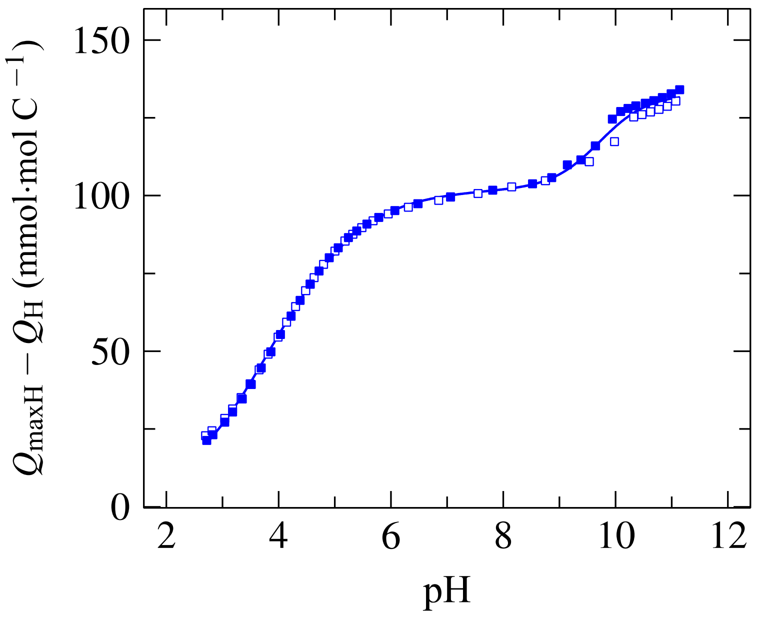

- Calculation of charge–pH curves and conditional parameters of the NICA model

The NICA isotherms originally developed for the description of the competitive binding of protons (H) and a concomitant metal ion 'M' are [1,2,3]:

$$
\begin{aligned}
Q_{\mathrm{H}} = {} & Q_{\mathrm{maxH,1}} \frac{\left(\bar{k}_{\mathrm{H,1}} c_{\mathrm{H,D}}\right)^{n_{\mathrm{H,1}}}}{\left(\bar{k}_{\mathrm{H,1}} c_{\mathrm{H,D}}\right)^{n_{\mathrm{H,1}}} + \left(\bar{k}_{\mathrm{M,1}} c_{\mathrm{M,D}}\right)^{n_{\mathrm{M,1}}}} \times \frac{\left[\left(\bar{k}_{\mathrm{H,1}} c_{\mathrm{H,D}}\right)^{n_{\mathrm{H,1}}} + \left(\bar{k}_{\mathrm{M,1}} c_{\mathrm{M,D}}\right)^{n_{\mathrm{M,1}}}\right]^{p_1}}{1 + \left[\left(\bar{k}_{\mathrm{H,1}} c_{\mathrm{H,D}}\right)^{n_{\mathrm{H,1}}} + \left(\bar{k}_{\mathrm{M,1}} c_{\mathrm{M,D}}\right)^{n_{\mathrm{M,1}}}\right]^{p_1}} + \\
& + Q_{\mathrm{maxH,2}} \frac{\left(\bar{k}_{\mathrm{H,2}} c_{\mathrm{H,D}}\right)^{n_{\mathrm{H,2}}}}{\left(\bar{k}_{\mathrm{H,2}} c_{\mathrm{H,D}}\right)^{n_{\mathrm{H,2}}} + \left(\bar{k}_{\mathrm{M,2}} c_{\mathrm{M,D}}\right)^{n_{\mathrm{M,2}}}} \times \frac{\left[\left(\bar{k}_{\mathrm{H,2}} c_{\mathrm{H,D}}\right)^{n_{\mathrm{H,2}}} + \left(\bar{k}_{\mathrm{M,2}} c_{\mathrm{M,D}}\right)^{n_{\mathrm{M,2}}}\right]^{p_2}}{1 + \left[\left(\bar{k}_{\mathrm{H,2}} c_{\mathrm{H,D}}\right)^{n_{\mathrm{H,2}}} + \left(\bar{k}_{\mathrm{M,2}} c_{\mathrm{M,D}}\right)^{n_{\mathrm{M,2}}}\right]^{p_2}}
\end{aligned}
\tag{S1}
$$

and

$$
\begin{aligned}
Q_{\mathrm{M}} = {} & \frac{n_{\mathrm{M,1}}}{n_{H,1}} Q_{\mathrm{maxH,1}} \frac{\left(\bar{k}_{\mathrm{M,1}} c_{\mathrm{M,D}}\right)^{n_{\mathrm{M,1}}}}{\left(\bar{k}_{\mathrm{H,1}} c_{\mathrm{H,D}}\right)^{n_{\mathrm{H,1}}} + \left(\bar{k}_{\mathrm{M,1}} c_{\mathrm{M,D}}\right)^{n_{\mathrm{M,1}}}} \times \frac{\left[\left(\bar{k}_{\mathrm{H,1}} c_{\mathrm{H,D}}\right)^{n_{\mathrm{H,1}}} + \left(\bar{k}_{\mathrm{M,1}} c_{\mathrm{M,D}}\right)^{n_{\mathrm{M,1}}}\right]^{p_1}}{1 + \left[\left(\bar{k}_{\mathrm{H,1}} c_{\mathrm{H,D}}\right)^{n_{\mathrm{H,1}}} + \left(\bar{k}_{\mathrm{M,1}} c_{\mathrm{M,D}}\right)^{n_{\mathrm{M,1}}}\right]^{p_1}} + \\
& + \frac{n_{\mathrm{M,2}}}{n_{\mathrm{H,2}}} Q_{\mathrm{maxH,2}} \frac{\left(\bar{k}_{\mathrm{M,2}} c_{\mathrm{M,D}}\right)^{n_{\mathrm{M,2}}}}{\left(\bar{k}_{\mathrm{H,2}} c_{\mathrm{H,D}}\right)^{n_{\mathrm{H,2}}} + \left(\bar{k}_{\mathrm{M,2}} c_{\mathrm{M,D}}\right)^{n_{\mathrm{M,2}}}} \times \frac{\left[\left(\bar{k}_{\mathrm{H,2}} c_{\mathrm{H,D}}\right)^{n_{\mathrm{H,2}}} + \left(\bar{k}_{\mathrm{M,2}} c_{\mathrm{M,D}}\right)^{n_{\mathrm{M,2}}}\right]^{p_2}}{1 + \left[\left(\bar{k}_{\mathrm{H,2}} c_{\mathrm{H,D}}\right)^{n_{\mathrm{H,2}}} + \left(\bar{k}_{\mathrm{M,2}} c_{\mathrm{M,D}}\right)^{n_{\mathrm{M,2}}}\right]^{p_2}}
\end{aligned}
\tag{S2}
$$

where $Q_{\mathrm{maxH},j}$ ($\mathrm{mol \cdot kg^{-1}}$) is the total amount of available proton binding sites within each distribution. In the previous equations $\bar{k}_{\mathrm{H},j}$ and $\bar{k}_{\mathrm{M},j}$ are the corresponding affinity constants of H and metal ions, respectively. The parameters $n_{i,j}$ ($0 < n_{i,j} \leq 1$) are ion-dependent values related to the non-ideality (stoichiometry) of the ion *i* and modal distribution *j*. The parameters p_j ($0 < p_j \leq 1$) account for the intrinsic heterogeneity of the modal distribution *j*, which is common for all the cations. The subindexes j = 1 and 2 represent the carboxylic and phenolic distribution of sites, respectively.

In absence of competing metal ions $c_{\mathrm{M,D}} = 0$, $Q_{\mathrm{M}} = 0$, and hence eq. (S1) reduces to the monocomponent isotherm for proton binding, which will be assumed hereafter:

$$Q_{\mathrm{H}} = Q_{\mathrm{maxH,1}} \frac{\left(\bar{k}_{\mathrm{H,1}} c_{\mathrm{H,D}}\right)^{m_1}}{1+\left(\bar{k}_{\mathrm{H,1}} c_{\mathrm{H,D}}\right)^{m_1}} + Q_{\mathrm{maxH,2}} \frac{\left(\bar{k}_{\mathrm{H,2}} c_{\mathrm{H,D}}\right)^{m_2}}{1+\left(\bar{k}_{\mathrm{H,2}} c_{\mathrm{H,D}}\right)^{m_2}} \quad \text{(S3)}$$

where $m_j = n_{\mathrm{H},j} \times p_j$. The local concentration of ion *i* (background salt ions and protons) in the gel phase, $c_{i,\mathrm{D}}$, is related to its concentration in bulk solution, c_i, by the Donnan factor χ^{z_i}:

$$c_{i,\mathrm{D}} = \chi^{z_i} c_i \quad \text{(S4)}$$

where z_i is the charge number of ion *i*.

The electrostatic charge of the DOM molecule, *q*, is related to the amount of proton (Q_{H}) binding through the mass balance:

$$q = Q_{\mathrm{H}} - Q_{\mathrm{maxH,1}} - Q_{\mathrm{maxH,2}} \quad \text{(S5)}$$

which implicitly assumes that all the DOM has been isolated in its neutral, fully protonated form.

The model assumes the electroneutrality condition given by [4,5]:

$$\frac{q}{V_{\mathrm{D}}} + \sum z_i \left(c_{i,\mathrm{D}} - c_i\right) = 0 \quad \text{(S6)}$$

where $q \leq 0$ and V_{D} represent the charge ($\mathrm{mol \cdot kg^{-1}}$) and volume ($\mathrm{L \cdot kg^{-1}}$) of the gel phase, respectively. Note that $V_{\mathrm{D}}\left(c_{i,\mathrm{D}} - c_i\right)$ represent the number of moles of ion *i* ($\mathrm{mol \cdot kg^{-1}}$) associated to (or excluded from) the Donnan phase by non-specific (electrostatic) binding.

The titrations were carried out with glass electrodes as the only ion-selective electrode. Thus, strictly speaking, only c_{H} is experimentally available, and so values of $c_{i \neq H}$ must be derived from mass-balance constraints. For this purpose, we define $c_{i,T}$ as the total (analytical) concentration of ion i in solution, so that

$$c_{\mathrm{H},T} = c_{\mathrm{H}} + \left\{ V_{\mathrm{D}} \left(c_{\mathrm{H,D}} - c_{\mathrm{H}} \right) + Q_{\mathrm{H}} \right\} c_{\mathrm{DOM}} \tag{S7}$$

$$c_{i,T} = c_i + \left\{ V_{\mathrm{D}} \left(c_{i,\mathrm{D}} - c_i \right) \right\} c_{\mathrm{DOM}} \quad ; \quad i \neq \mathrm{H} \tag{S8}$$

where c_{DOM} is the concentration of DOM in $\mathrm{kg{\cdot}L^{-1}}$.

In the case of background ions other than protons $Q_{i \neq \mathrm{H}}$ is assumed negligible.

In this way, the following relationship (or “macroscopic” charge balance) is straigthforwardly derived from eqs. (S6) and (S8):

$$q' \cdot c_{\mathrm{DOM}} + c_H - \frac{K_w}{c_{\mathrm{H}}} + \sum_{i \neq \mathrm{H,OH}} z_i c_{i,T} = 0 \tag{S9}$$

where q' is the “apparent” charge of DOM, defined as:

$$q' = q + V_{\mathrm{D}} \left[\left(\chi - 1 \right) c_{\mathrm{H}} - \left(\frac{1}{\chi} - 1 \right) \frac{K_w}{c_{\mathrm{H}}} \right] \tag{S10}$$

and K_{w} is the stoichiometric water dissociation constant.

Eq. (S9) is used to obtain the apparent charge curves (pH, q') from the experimental titration data.

On the other hand, the values of χ can be calculated from eq. (S6), taking into account that the bulk solution compartment is also electroneutral, *i.e.*: $\sum z_i c_i = 0$, which leads to:

$$\frac{q}{V_{\text{D}}}+\sum z_i \chi^{z_i} c_i = 0 \qquad \text{(S11)}$$

Substitution of eqs. (S7), (S8) and (S10) in the latter relationship yields:

$$\frac{q'}{V_{\text{D}}}+c_{\text{H}}-\frac{K_w}{c_{\text{H}}}+\sum_{i\neq \text{H,OH}} \frac{z_i \chi^{z_i} c_{i,T}}{1+V_{\text{D}} c_{\text{DOM}} \left(\chi^{z_i}-1\right)} = 0 \qquad \text{(S12)}$$

which can be solved to obtain the value of χ, once V_{D} is known. At intermediate pH values and not too low ionic strengths, the assumption of $V_{\text{D}} c_{\text{DOM}} \approx 0$ leads to:

$$\frac{q}{V_{\text{D}}}+\sum_{i\neq \text{H,OH}} z_i \chi^{z_i} c_{i,T} = 0 \qquad \text{(S13)}$$

which is the expression commonly used in literature.

In those cases where the polyelectrolytic effect is not explicitly taken into account (i.e. because it is negligible and/or only data at one single value of ionic strength is available), the electrostatic contribution to the binding can be disregarded (which is equivalent to assume the Donnan factor $\chi^{z_i} \approx 1$) and, therefore the bulk concentrations c_i are used instead of $c_{i,\text{D}}$ so that eq. (S3) reduces to:

$$Q_{\text{H}} = Q_{\text{maxH,1}} \frac{\left(K_{\text{H,1}} c_{\text{H}}\right)^{m_1}}{1+\left(K_{\text{H,1}} c_{\text{H}}\right)^{m_1}} + Q_{\text{maxH,2}} \frac{\left(K_{\text{H,2}} c_{\text{H}}\right)^{m_2}}{1+\left(K_{\text{H,2}} c_{\text{H}}\right)^{m_2}} \qquad \text{(S14)}$$

which corresponds to eq. (1) in the main manuscript. Note that now $K_{\text{H},j}$ and m_j correspond to *conditional* values which are only valid for the ionic strength of the experiments. In the present manuscript, the experimental ionic strength chosen (0.7 M) is high enough so that the electrostatic effects are negligible [6], and hence the difference between conditional and *intrinsic* values should be negligible.

- Comparison with a generic fulvic acid from Milne *et al*. 2001 [3]:

In the standard (or classical) implementation of the NICA–Donnan model, an empirical relationship was used for V_D based on an apparent log-log relationship with ionic strength, I, as reported by Benedetti et al. [7]:

$$\log V_{\mathrm{D}} = b\left(1 - \log I\right) - 1 \tag{S15}$$

This relationship implies that V_D depends only on ionic strength and not on the macromolecular charge density. This is the model most commonly used in literature and it has been applied in the derivation of the so-called "generic" NICA–Donnan model parameters [3,8]. However, the empirical nature of eq. (S15) yields master curves (intrinsic charge curves) that strongly deviate from titrations at high ionic strength [9,10]. In our case, the average (generic) values of the intrinsic parameters $Q_{\mathrm{maxH,j}}$, $\overline{k}_{\mathrm{H},j}$ and m_j, as well as the average value of b in eq. (S15), reported by Milne et al. [3] for fulvic acids were used to "generate" a theoretical charge curve at I=0.7 M, which was later fitted to the conditional isotherm eq. (S14) (eq. (1) in the main manuscript) in the same way as the experimental charge curves obtained with the SPE–DOM samples. The resulting conditional values for a generic fulvic acid were used for comparison purposes as discussed in the manuscript.

References

(1) Kinniburgh, D. G.; van Riemsdijk, W. H.; Koopal, L. K.; Borkovec, M.; Benedetti, M. F.; Avena, M. J. Ion Binding to Natural Organic Matter: Competition, Heterogeneity, Stoichiometry and Thermodynamic Consistency. *Colloids Surfaces A Physicochem. Eng. Asp.* **1999**, *151* (1–2), 147–166. https://doi.org/10.1016/S0927-7757(98)00637-2.

(2) Koopal, L. K.; van Riemsdijk, W. H.; de Wit, J. C. M.; Benedetti, M. F. Analytical Isotherm Equations for Multicomponent Adsorption to Heterogeneous Surfaces. *J. Colloid Interface Sci.* **1994**, *166* (1), 51–60. https://doi.org/10.1006/jcis.1994.1270.

(3) Milne, C. J.; Kinniburgh, D. G.; Tipping, E. Generic NICA-Donnan Model Parameters for Proton Binding by Humic Substances. *Environ. Sci. Technol.* **2001**, *35* (10), 2049–2059. https://doi.org/10.1021/es000123j.

(4) Benedetti, M. F.; Van Riemsdijk, W. H.; Koopal, L. K.; Kinniburgh, D. G.; Gooddy, D. C.; Milne, C. J. Metal Ion Binding by Natural Organic Matter: From the Model to the Field. *Geochim. Cosmochim. Acta* **1996**, *60* (14), 2503–2513. https://doi.org/10.1016/0016-7037(96)00113-5.

(5) Kinniburgh, D. G.; Milne, C. J.; Benedetti, M. F.; Pinheiro, J. P.; Filius, J.; Koopal, L. K.; Van Riemsdijk, W. H. Metal Ion Binding by Humic Acid: Application of the NICA-Donnan Model. *Environ. Sci. Technol.* **1996**, *30* (5), 1687–1698. https://doi.org/https://doi.org/10.1021/es950695h.

(6) Lodeiro, P.; Rey-Castro, C.; David, C.; Achterberg, E. P.; Puy, J.; Gledhill, M. Acid-Base Properties of Dissolved Organic Matter Extracted from the Marine Environment. *Sci. Total Environ.* **2020**, *729*, 138437. https://doi.org/10.1016/j.scitotenv.2020.138437.

(7) Benedetti, M. F.; vanRiemsdik, W. H.; Koopal, L. K. Humic Substances Considered as a Heterogeneous Donnan Gel Phase. *Environ. Sci. Technol.* **1996**, *30* (6), 1805–1813. https://doi.org/https://doi.org/10.1021/Es950012y.

(8) Milne, C. J.; Kinniburgh, D. G.; van Riemsdijk, W. H.; Tipping, E. Generic NICA−Donnan Model Parameters for Metal-Ion Binding by Humic Substances. *Environ. Sci. Technol.* **2003**, *37* (5), 958–971. https://doi.org/10.1021/es0258879.

(9) Koopal, L.; Tan, W.; Avena, M. Equilibrium Mono- and Multicomponent Adsorption Models: From Homogeneous Ideal to Heterogeneous Non-Ideal Binding. *Adv. Colloid Interface Sci.* **2020**, *280*, 102138. https://doi.org/10.1016/j.cis.2020.102138.

(10) Town, R. M.; van Leeuwen, H. P.; Duval, J. F. L. Rigorous Physicochemical Framework for Metal Ion Binding by Aqueous Nanoparticulate Humic Substances: Implications for Speciation Modeling by the NICA-Donnan and WHAM Codes. *Environ. Sci. Technol.* **2019**, *53* (15), 8516–8532. https://doi.org/10.1021/acs.est.9b00624.
